# Syndrome in question*

**DOI:** 10.1590/abd1806-4841.20153818

**Published:** 2015

**Authors:** Priscila Regina Orso Rebellato, Camila Makino Rezende, Eveline Roesler Battaglin, Brunno Zeni de Lima, Jose Fillus Neto

**Affiliations:** 1 Faculdade Evangélica do Paraná (Fepar) - Curitiba (PR), Brazil

**Keywords:** Edema, Erythema, Face

## Abstract

Morbihan Syndrome is a rare entity with unknown etiology. It is clinically characterized by chronic erythematous edema on the face - especially in the middle and upper third of the face - and creates abnormal facial contours that are initially intermitent but become permanent with the development of the syndrome. The histopathology is nonspecific and its therapy is a major challenge due to poor response to the various treatment options. We present the case of a male patient with a five-month-history of disease.

## CASE REPORT

**Figure 1:**
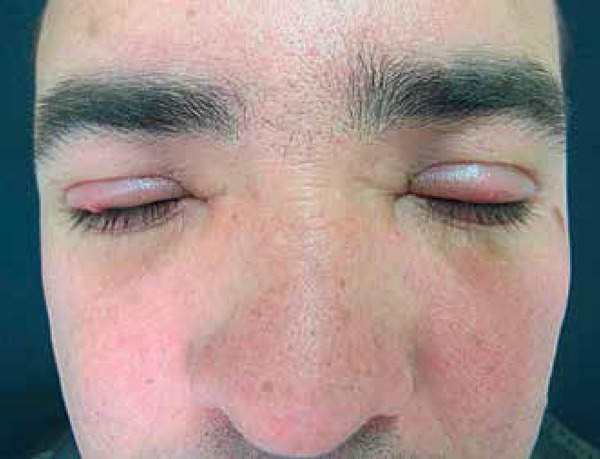
Periorbital edema, larger on the upper eyelid, with superimposed erythematous papules (Figure 1).

A 38-year-old male patient presented with a periorbital indurated edema, which had been progressively increasing for 5 months. The edema was painless, larger on the upper eyelid, remained constant throughout the day and coincided with the intermittent appearance of superimposed erythematous papules (Figure 1). The patient denied itching and had no eye or muscle symptoms. He had no previous skin diseases, comorbidities or addictions, and an unremarkable family history. The patient was in good general condition. There were neither changes in other organs nor systemic symptoms.

Full blood count, renal function, VHS, proteinuria, FAN, anti-JO1, complement dosages and orbit CT were within normality.

**Figure 2:**
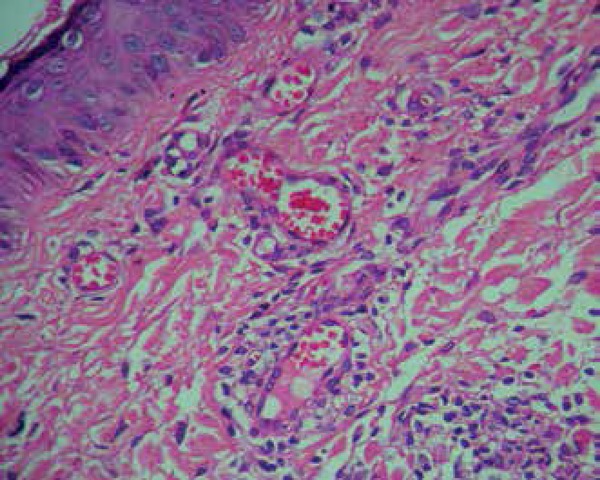
Intact basal layer. Superficial dermis with vessel ectasia, mild fibrosis, edema and mild inflammatory infiltrate of mononuclear cells (400x HE)

**Figure 3:**
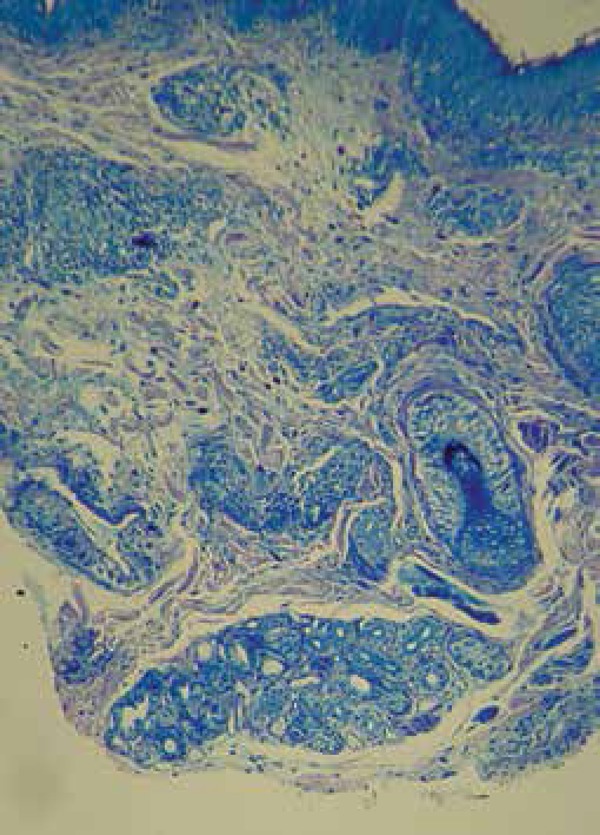
Evident increase in the number of mast cells and increased vascular dilatation (100x Giemsa stain)

Histopathology showed a preserved epidermis, chronic perivascular infiltration of the papillary and middle dermis, dilated vessels and an increased number of mast cells (Figures 2 and 3). These findings suggested the diagnosis of Morbihan Syndrome.

Treatment was initiated with minocycline and hydroxyzine. Nevertheless, as the patient showed no improvement after 3 months, treatment was changed to isotretinoin 20 mg/day for 6 months, combined with ketotifen 1 mg/day for the first 2 months. At the end of this period, the patient showed good clinical improvement with no local skin sequelae.

## DISCUSSION

Morbihan syndrome is characterized by gradual erythema and solid edema mainly in the upper two-thirds of the face. It may affect the forehead, glabella, eyelids, nose and chin.^1,2^ It evolutes from initial intermittent outbreaks that later become persistent and lead to a solid and permanent infiltration of the skin, causing its bulking and altering the facies. Patients usually report subjective symptoms due to the disfigurement of the facial contour, which may affect them psychologically and cosmetically. It leaves the patient in good general condition and there is no systemic involvement.^3^

Its etiology is uncertain. It is assumed that triggering factors such as immune contact urticaria would produce an increase in lymphatic load that would exceed the drainage capacity of the lymph, leading to a persistent facial swelling.^3^ Other explanations include chronic inflammation due to acne and rosácea causing structural damage to blood and lymph vessels^1,4^, and a post-infectious inflammation cause by recurrent simple herpes outbreaks.^2^

The histopathology is nonspecific.^5^ A mild edema in the mid and deep dermis is found, along with ectatic lymph vessels and la ymphohistiocytic perivascular and perifollicular infiltrate.^1,3^ An increased number of mast cells^3^ and granulomas^1,5^ may be found. In the case of a previous history of acne or rosácea, hyperplasia of sebaceous glands may be observed.^5^

Laboratory and imaging tests are normal and do not help in the diagnosis.^2,5^

The differential diagnoses include variants of the Melkersson-Rosenthal syndrome, dermatomyositis, lupus erythematosus, lacrimal gland lymphoma, sarcoidosis, chronic contact dermatitis, among others.^3^ One could think of the blepharo-naso-facial syndrome as another differential diagnosis. Nevertheless, this syndrome usually has its onset at puberty, has a recurrent character and may lead to chronic changes in eyelid support structures.

Systemic steroids, radiation therapy, antibiotics, thalidomide and clofazimine have already been used in the treatment of this syndrome, but have shown poor response rates.^3^ Isotretinoin alone or in combination with ketotifen appears to be effective. More recent studies state that its use at high doses and for a long period of time can lead to good results.^1,5^ Our patient improved after treatment with isotretinoin combined with ketotifen, despite of the use at low doses and for a short period of time. Facial lymphatic drainage can be used as a supporting treatment^4^, and contact with irritants or allergens that possibly trigger the disease should be avoided.^3,4^
